# Scientific trends on research on denture stomatitis based on Scopus database: A bibliometric analysis

**DOI:** 10.4317/jced.60249

**Published:** 2023-03-01

**Authors:** Beenish-Fatima Alam, Talha Nayab, Amr S. Bugshan, Mohammed M. Gad, Erum Khan, Saqib Ali

**Affiliations:** 1 BDS, MSc, MFDS RCSEd. Associate Professor, Department of Oral Biology, Bahria University Medical and Dental College, Karachi, Pakistan; 2 BDS, MSc. Assistant Professor, Department of dental materials science, Sindh Institute of Oral Health Sciences, Jinnah Sindh Medical University, Karachi, Pakistan; 3 BDS, PhD. Assistant Professor Department of Biomedical Dental Sciences, College of Dentistry, Imam Abdulrahman Bin Faisal University, Dammam, Saudi Arabia; 4 BDS, MSc,. Lecturer, Department of Substitutive Dental Sciences, College of Dentistry, Imam Abdulrahman Bin Faisal University, Dammam, Saudi Arabia; 5 BDS, MSc. Director, CODE-M, Center of Dental Education and Medicine, Karachi, Pakistan; 6 BDS, MSc, MFDS RCPS. Lecturer, Department of Biomedical Dental Sciences, College of Dentistry, Imam Abdulrahman Bin Faisal University, Dammam, Saudi Arabia

## Abstract

**Background:**

Denture stomatitis is a clinical condition that affects people who wear removable maxillary dentures. It causes redness, soreness, and erythema and ultimately affects the general condition of the patient. The objective of this study was to analyze the leading countries, journals, organizations, and authors and the frequently used keywords associated with denture stomatitis.

**Material and Methods:**

A bibliometric analysis of publications indexed in the Scopus database was conducted, and the article titles, abstracts, and keywords were analyzed using the VOSviewer software. Denture stomatitis-related publications from 1960 to 2021 were collected. This study included only research papers published in English with “article” as the paper type and dentistry as the subject area.

**Results:**

Data from a total of 461 articles and 10 different journals were obtained. The papers were published in 64 different countries. Brazil and the United States of America were the top contributing countries, and the University of Sydney was the leading organization. Papers published in the Journal of Oral Rehabilitation received the highest number of citations, while author Gordon Ramage from the University of Glasgow received the highest number of citations.

**Conclusions:**

The bibliometric analysis revealed that the number of denture stomatitis-related publications indexed in the Scopus database is increasing globally. Since 2007, there has been an increase in research interest regarding denture stomatitis, with more publications from several countries expected to be published in different journals.

** Key words:**Bibliometric analysis, denture, candida, VOSviewer, maxilla.

## Introduction

Denture stomatitis (DS) is a common oral condition that affects people who wear removable dental prostheses (1). DS is caused by various factors, including poor oral hygiene, trauma caused by poorly fabricated dental prostheses, plaque accumulation, and resin porosity, all of which contribute to increased susceptibility to infection (2). This condition frequently affects around 65% of maxillary complete denture wearers. Similarly, the palatal mucosa is commonly affected but remains asymptomatic (3).

Clinically, this condition is characterized by varying degrees of redness and edema, and it sometimes occurs with mucosal petechial hemorrhage localized to the tissue-contacting surface of removable dental prostheses (2,3). However, the role of *Candida* infection in the development of DS remains controversial, and other microorganisms living beneath the denture may also contribute to its development (3,4). DS can be caused by other factors, such as smoking, nutritional deficiency, various medical disorders, denture status, denture cleaning habits, and continuous wearing of dentures, in addition to infection (5,6).

New treatment strategies, including the use of various natural products (7), photodynamic therapy (8), and the use of nanomaterials, have been implemented for the effective management of DS (9). Nevertheless, the most effective treatment for DS is to provide denture wearers oral hygiene instructions for maintaining dental prostheses and to use topical antifungal agents (10).

Many papers have been published in the past in light of the detrimental effects of DS on the oral and general health of individuals. In recent years, these scientific publications have increased in relation to the number of journals, resulting in an increase in the number of published papers. Thus, potential investigators and academicians must find the most useful article in the literature. It is also important to investigate this growth to assess its consequences and influence on research (11). One way to assess this growth is to conduct a bibliometric analysis, which entails using statistical means to analyze the research to identify the advancement and development of a particular subject (12).

Bibliometric analyses have been conducted in various fields of dentistry, including minimally invasive dentistry (13), early childhood caries (12), endodontic microbiology (14), dental stem cells (15), and oral submucous fibrosis (16). However, no bibliometric analysis of DS has yet been documented. Therefore, this study aimed to determine the leading authors, organizations, countries, articles, journals, and keywords associated with published DS-related articles indexed in the Scopus database.

## Material and Methods

-Search strategy and keywords

On November 9th, 2021, Elsevier’s Scopus database was searched for all DS-related articles published during a 61-year period from 1960 to 2021 in order to perform the bibliometric analysis. The electronic search included the research topic, which further included the titles, abstracts, and keywords. To retrieve results from the Scopus database, the keyword “denture stomatitis” was used for the search.

-Inclusion and exclusion criteria

**Figure 1:**
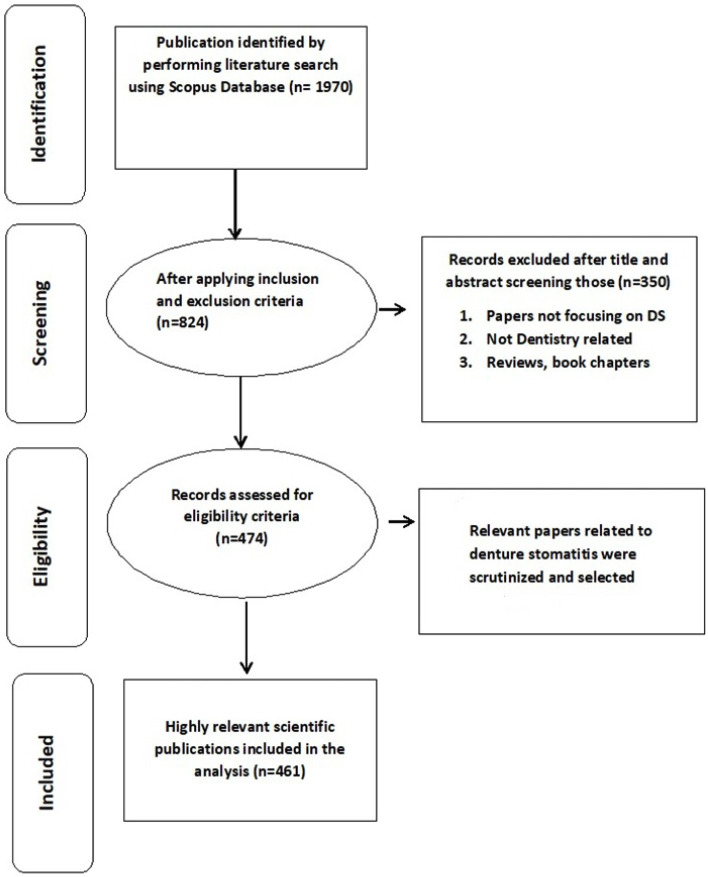
Four-phase flow diagram.

Exclusion and inclusion criteria were applied to further narrow the results of the search for relevant DS-related papers. The inclusion criteria for this study were as follows: a) papers published in English language, b) “original article” as the paper type, and c) dentistry as the subject area. The exclusion criteria were (a) papers not focusing on denture stomatitis; b) other paper types, this includes reviews, case studies, and book chapters; and c) papers from fields other than dentistry (Fig. 1).

-Data analysis

To identify the leading countries, authors, journals, and institutes as well as the highly cited papers and frequently used keywords, all articles were selected and exported from Scopus in comma-separated values (CSV) format. The search results were saved and then transferred as tab-delimited files, which were subsequently analyzed using VOSviewer (v1.6.16; Center for Science and Technology Studies, Leiden University), a bibliometric software program (17-19). The VOSviewer software was used to generate collaborative networks for different variables and keywords. The dimensions of the bubbles in the generated maps indicated the number of publications, while the distance between two bubbles indicated the similarity between the two items. The color of each bubble had distinct meanings in each visualization. Keywords with the most occurrences were selected, and visualization maps were generated.

## Results

A total of 824 articles published in 88 journals were retrieved from the Scopus database. Owing to the presence of one or more of the study exclusion criteria, 363 articles and 78 journals were excluded. Based on the inclusion and exclusion criteria of the study, 461 articles published in 10 journals between 1960 and 2021 were deemed eligible for further review.

**Figure 2:**
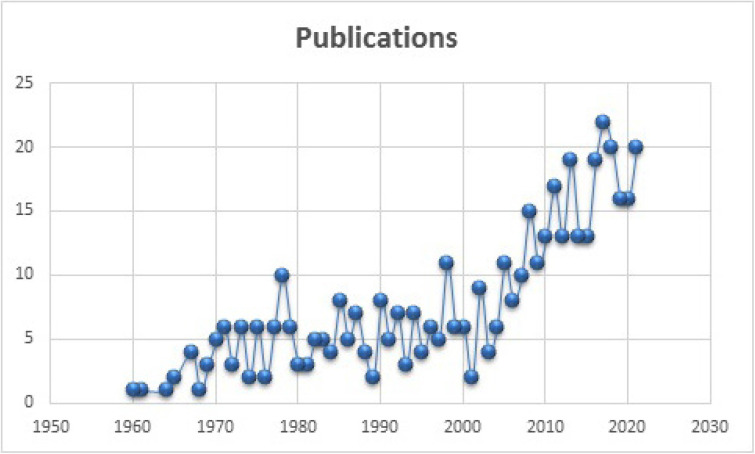
Research trend of publications from 1960 to 2021.

Figure 2 demonstrates that research on DS began in the early 1960s. However, since 2007 onwards there has been a surge in interest in the domain, and 10 papers were published in 2007. The highest number of publications occurred in 2017 and 2018, with 22 and 20 articles published, respectively. However, 20 papers were published as of November 2021, and this number is expected to increase in the upcoming years (Fig. 2).

Table 1 shows the leading countries with the highest number of DS-related articles published by their researchers. A total of 64 countries published DS-related papers, with only 10 countries publishing at least 14 articles. Brazil was the leading country with the highest number of published articles [76], followed by the United States [66], the United Kingdom [52], and India [24] (Table 1).

**Table 1:**
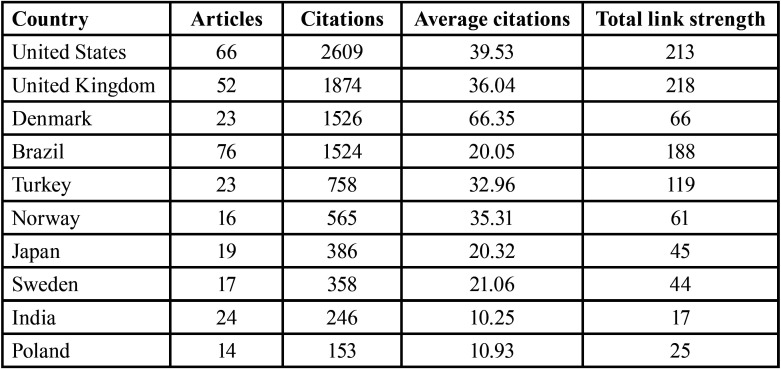
Top contributing countries.

The United States had the highest number of citations [2609], with an average of 39.53 citations per article. The United Kingdom came in second [1874 citations], with an average of 36.04 citations per paper. Denmark and Brazil had 1526 and 1524 citations, respectively. The United Kingdom, the United States, Brazil, and Turkey were among the highly collaborative countries that had the highest total link strength [218, 213, 188, and 119, respectively] (Table 1).

The University of Sydney in Australia was the top contributing organization [10 articles] with the highest number of citations [1128] and an average of 112.8 citations per article. The Royal Dental College in Denmark [9 articles] came in second, with 691 citations [the average number of citations per article was 76.8], and the University of New South Wales in Australia, which published 3 articles with 357 citations, had the highest average number of citations per article [119] (Table 2).

**Table 2:**
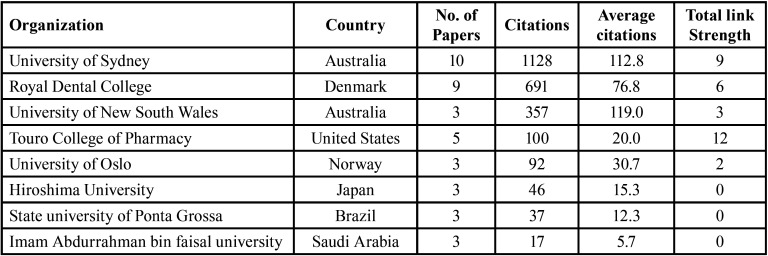
Top contributing organisations.

Table 3 lists the top contributing journals according to the highest number of published articles. In this domain, 10 out of 88 journals published 12 articles. The Journal of Prosthetic Dentistry published the highest number of papers [57], followed by the Journal of Gerodontology, Acta Odontologica Scandinavica, and the Journal of Oral Rehabilitation [29, 27, and 25, respectively].

**Table 3:**
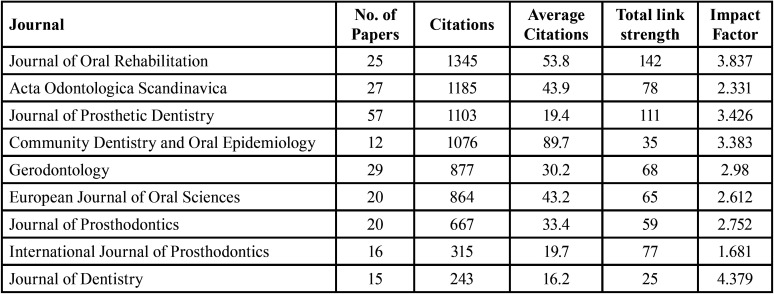
Top contributing journals.

The Journal of Oral Rehabilitation received the highest number of citations [1345; the average number of citations per article was 53.8], followed by Acta Odontologica Scandinavica [1185 citations; the average number of citations per article was 43.9]. Although the Journal of Prosthetic Dentistry had the highest number of published articles with 1103 citations, it had only an average of 19.4 citations per article. The Journal of Oral Rehabilitation had the highest total link strength [142], followed by the Journal of Prosthetic Dentistry [111], Acta Odontologica Scandinavica [78], and the International Journal of Prosthodontics [77] (Table 3).

Table 4 lists the top 13 authors out of 1554 authors who have contributed in research related to DS. Ingar Olsen had the highest number of published articles [10] but received only 356 citations [an average of 35.60 citations per article]. However, Gordon Ramage [who published 6 articles] and E. Budtz-Jørgensen [who published 7 articles] received the highest average number of citations per article [99.83 and 79.57, respectively). Zvi G. Loewy had the highest total link strength [37], followed by Gordon Ramage [31], Carlos Eduardo Vergani [29], and Jeremy Bagg [24] (Table 4).

**Table 4:**
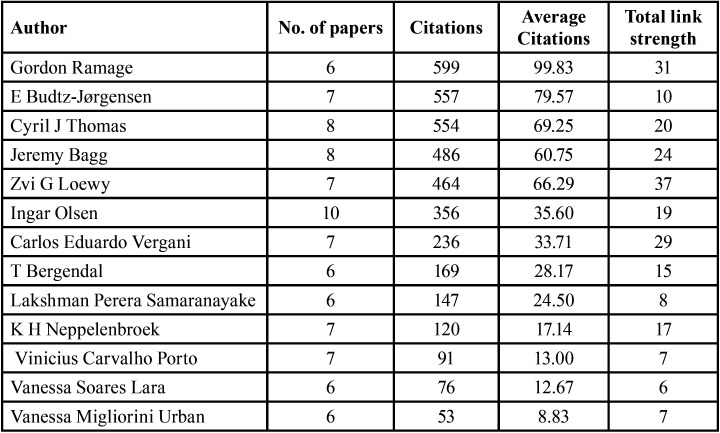
Leading authors.

**Figure 3:**
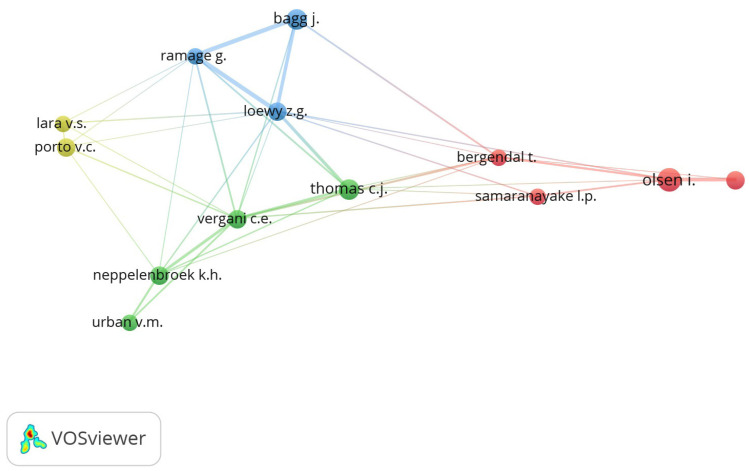
Collaborative network among different authors.

Figure 3 depicts the collaborative network of numerous authors who have conducted research on DS. Gordon Ramage, who received the most citations for his papers, has collaborated with Jeremy Bagg, Zvi G. Loewy, and Cyril J. Thomas.

 Table 5 lists the 11 most cited articles out of the 461 articles with more than 160 citations. Linda Gendreau and Zvi G. Loewy’s article received the highest number of citations [324] (20), followed by E. Budtz-Jørgensen’s article [303 citations], and Ramage *et al*.’s paper [285 citations].

**Table 5:**
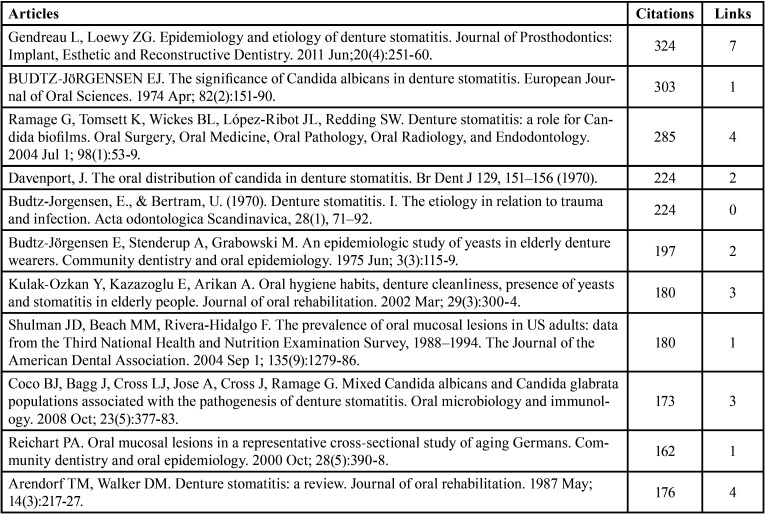
Highly cited articles.

**Figure 4:**
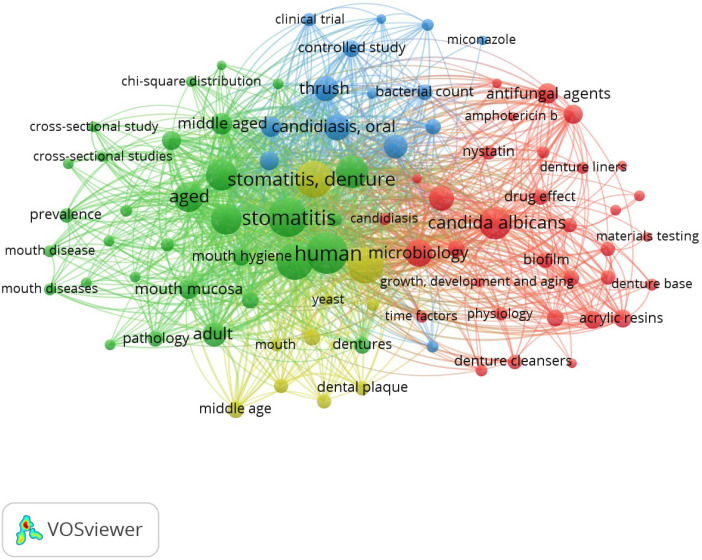
Frequently used keywords.

Figure 4 displays the frequently used keywords. Out of 2248 keywords, 88 most frequently used keywords [20 times or more] were stomatitis [293], stomatitis denture [254], denture [231], *Candida albicans* [188], candidiasis, oral [130].

## Discussion

The number of research publications and journals that are publishing DS-related papers is increasing rapidly owing to recent advancements in treatment strategies for DS. In this study, a bibliometric analysis was conducted to study the literature on DS in order to determine the areas of focus and the areas with more potential and prospects. This will assist researchers, clinicians, and academicians in providing precise research directions.

The major findings of this study revealed that most published dental-related articles originated from Brazil and the United States, which is consistent with previous bibliometric analysis results (21). The United States had the highest number of citations, followed by the United Kingdom. This could be because these countries, particularly the United States, have a well-developed National Institute of Dental and Craniofacial Research (NIDCR), which is generally responsible for providing research funds to both the public and private sectors (13).

The University of Sydney in Australia was the leading organization that published the highest number of DS-related articles. This is consistent with the findings of the bibliometric analysis conducted by Weng *et al*. (22). The Royal Dental College in Aarhus published the second-highest number of DS-related articles.

An interesting finding of this study was that most of the articles were published in the most influential and pertinent journals, all of which have a high impact factor, indicating that papers published in these journals will receive more attention and citations (23). The Journal of Prosthetic Dentistry, which has an impact factor of 3.426, published 57 articles and received 1103 citations, followed by the Journal of Gerodontology, which has an impact factor of 2.98, published 29 articles, and received 877 citations. Interestingly, the Journal of Oral Rehabilitation published 25 articles and received the highest number of citations [1345], followed by Acta Odontologica Scandinavica. Thus, this publication trend identifies the influence of the impact factor of a journal and the predisposition of authors to cite an article published in a high-impact factor journal in a particular field.

Ingar Olsen from the University of Oslo in Norway was the most prominent first author who contributed significantly and whose papers were identified as the top-cited articles, with 10 published articles receiving 356 citations, followed by Cyril Thomas from the University of Sydney in Australia [published 8 articles and received 554 citations], and Jeremy Bagg from Glasgow University in the United Kingdom [published 8 articles and received 486 citations]. Interestingly, Gordon Ramage published only 6 articles but received the highest number of citations [599]. This is because the influence and quality of a research topic play a significant role in the domains of research and clinical practice.

Linda Gendreau published an article that received the highest number of citations [324]. In this review, literature search was conducted using the PubMed database to find appropriate articles that focused on the prevalence and causative factors of DS and the importance of using the appropriate dental material in managing this condition (20). E. Budtz-Jörgensen’s article, which discussed the occurrence, causative factors, distinctive features, and diagnostic findings related to the effect of *Candida* on denture wearers, received the second-highest number of citations [303] (24). Gordon Ramage’s article on the analysis of the biofilms of DS patients using SEM was the third most cited article. These biofilms tended to adhere to the denture’s cracks and other faults propagated within the denture, and they were also found to be resistant to antifungal treatment (25).

A keyword analysis is a significant bibliometric indicator. It can detect potentially trending research topics and provide a clear aspect of research hotspot analysis. The appropriate keywords can be used to accurately detect a detailed grouping and exploration of research areas, as they retrieve pertinent results. These keywords, when used in publications, tend to act as “codes” that aid in finding the relevant literature. Keyword exploration detects current and historical trending research domains (26). The most commonly used keywords were stomatitis, denture stomatitis, *Candida albicans*, and candidiasis.

Although this study provided relatively all-round visualized analyses of information related to publications on DS, there were still limitations. First, articles published in languages other than English were excluded. Moreover, some recently published articles may have been missed because only the Scopus database was used for the analysis. Additionally, if bibliometric data change over time, a different conclusion may be drawn.

## Conclusions

The bibliometric analysis revealed that the number of published DS-related articles indexed in the Scopus database is increasing globally. Interest in research related to denture stomatitis has continued to increase, with more publications in various countries, journals, and subjects. Brazil and the United States were the largest contributors to research in relation to denture stomatitis and were leaders in this field. The Journal of Oral Rehabilitation published the most cited articles in the field, and Gordon Ramage *et al*. received the highest number of citations for their published articles.
